# Neuronal Voltage Gated Potassium Channels May Modulate Nitric Oxide Synthesis in Corpus Cavernosum

**DOI:** 10.3389/fphar.2017.00297

**Published:** 2017-05-26

**Authors:** Amira M. Senbel, Heba M. Abd Elmoneim, Fouad M. Sharabi, Mahmoud M. Mohy El-Din

**Affiliations:** Department of Pharmacology and Toxicology, Faculty of Pharmacy, Alexandria UniversityAlexandria, Egypt

**Keywords:** nitric oxide, potassium channels, erection, corpus cavernosum, voltage- gated potassium channels, 4-amino pyridine

## Abstract

Potassium channels (K^+^Ch) in corpus cavernosum play an important role in the regulation of erection. Nitric oxide (NO) acts through opening of K^+^Ch leading to hyperpolarization and relaxation.

**Aim :** This study aims to update knowledge about the role of voltage-gated K^+^Ch (K_V_) channels in erectile machinery and investigate their role in the control of NO action &/or synthesis in the corpus cavernosum.

**Methods :** Tension studies using isolated rabbit corpus cavernosum (CC) strips and rat anococcygeus muscle were conducted. Results are expressed as mean ± SEM.

**Results :** Electric field stimulation (EFS, 2–16 Hz) evoked frequency-dependent relaxations of the PE (phenylephrine)-precontracted CC strips. At 2 Hz, EFS-induced relaxation amounted to 73.17 ± 2.55% in presence 4-AP (10^−3^ M) compared to 41.98 ± 1.45% as control. None of the other selective K^+^Ch blockers tested inhibited EFS-induced relaxation. 4-AP (10^−3^M) significantly attenuated ACh-induced relaxation of rabbit CC where dose-response curve was clearly shifted upward, and attenuated SNP- induced relaxation, for example, to 49.28 ± 4.52% compared to 65.53 ± 3.01% as control at 10^−6^ M SNP. The potentiatory effect of 4-AP on EFS was abolished or reversed in presence of N^*G*^-nitro-L-arginine (L-NNA, non-selective nitric oxide synthase inhibitor, 10^−5^M, and 2 × 10^−4^M). Same results were observed in rat anococcygeus muscle which is a part of the erectile machinery in rats.

**Conclusion :** This study provides evidence for the presence of prejunctional voltage-gated K^+^Ch in CC, the blockade of which may increase the neuronal synthesis of NO.

## Introduction

Penile erection is the end result of a complex neuro-vascular process in which nerves, endothelium of sinusoids and blood vessels, and smooth muscle cells in the target organ are involved (Andersson, 2011). Penile erection is achieved by dilatation of penile arteries and relaxation of the trabecular smooth muscles located in the corpus cavernosum (CC) with subsequent compression of penile veins (Gratzke et al., 2010). The main mediator of this smooth muscle cell relaxation in the penis is nitric oxide (NO), which is synthesized by eNOS and nNOS, in nitrergic nerves, endothelial cells, and cavernosal smooth muscle cells. The increased NO production induces activation of sGC, increased cGMP levels, and activation of PKG (Musicki et al., 2009). Protein kinase G phosphorylates several key target proteins, including ion channels, ion pumps, and enzymes; all involved in the control of intracellular calcium level. Among these target ion channels are K^+^ channels (Lin et al., 2005; Prieto, 2008). Phosphorylation of K^+^ channels by PKG leads to their activation with subsequent hyperpolarization and relaxation of corporal smooth muscle cells (Christ, 2000, 2002; Lee, 2000; Archer, 2002).

Four major types of K^+^ channels are expressed in smooth muscles: large conductance Ca^2+^-activated (BK_Ca_), ATP-sensitive (K_ATP_), inward rectifier (Kir) and voltage-gated (K_V_) (Stott et al., 2014). (BK_Ca_) channels have been characterized in the CC smooth muscle of several species including rabbits, where they are thought to be important regulators of smooth muscle tone (Fan et al., 1995; Wang et al., 2000; Malysz et al., 2001; Werner et al., 2005; Hannigan et al., 2016). Electrophysiological characterization has demonstrated the expression of K_ATP_ channels in corporal smooth muscle cells, which induce muscle relaxation upon channel activation (Holmquist et al., 1990; Lee et al., 1999; Insuk et al., 2003). On the other hand, activation of Kir channels have been implicated in mediating part of the relaxant response to *Schisandra chinensis* extract in rabbit CC (Han et al., 2012). Furthermore, the endothelium- independent relaxation of rat CC induced by taurine (Dalaklioglu-Tasatargil, 2013) and resveratrol (Dalaklioglu and Ozbey, 2014) was proved to depend in part on Kir channels activation. Evidence for the presence of K_V_ channels in rabbit CC have been provided by Malysz et al. (2001), where the authors reported at least two types of smooth muscle cells in the CC, one with predominant K_V_ channels and the other with predominant (BK_Ca_) channels. In addition, the K_*V*_ channels appeared to be important for the regulation and control of the membrane potential in cavernous smooth muscle cells (Malysz et al., 2001, 2002).

Voltage-gated K^+^ channels comprise the largest and most diverse family among human K^+^ channels, with 12 known subfamilies (KV1–KV12) (Wulff et al., 2009; Lily Jan et al., 2016). These channels are generally activated by depolarization, therefore they tend to play roles in repolarizing membranes in nerve and muscle cells, thus controlling action potential frequency and duration. In vascular smooth muscle cells, vasoconstrictors stimulate the G-protein-coupled receptors, which cause phospholipase C/ diacylglycerol activation and consequently, activate PKC that induce K_V_ channel inhibition. Vasodilators stimulate the production of cAMP and cGMP, which in turn increase K_V_ channel activity through the stimulation of PKA and PKG respectively (Ko et al., 2010). K_V_ channels expression has been detected in vascular smooth muscle cells, where they limit membrane depolarization, vasoconstriction, and maintain resting vascular tone (Ko et al., 2008). The major K_V_ channels expressed in the vasculature are KV1.2, KV1.5, KV2.1, and KV7.4/7.5. Their distribution varies considerably with vascular bed, and there is some controversy over their relative contribution to the regulation of the resting membrane potential (Humphries and Dart, 2015). As for the potential role of K_V_ channels on nerve cells, limited data is available from peripheral system. In 2016, blockade of K_V_ channels by 4-aminopyridine was proved to increase GABA and glutamate release in midbrain (Li et al., 2016). Same observation was reported in guinea pig cerebrocortical synapses by Tibbs et al. (1989) and by Schnee and Brown (1998) in rat hippocampus (Schnee and Brown, 1998). In general, altered K_V_ channel expression and function is related to pathophysiological conditions such as systemic arterial hypertension, hypoxic pulmonary vasoconstriction, pulmonary arterial hypertension (Ko et al., 2010) and Parkisonism (Luca and Singer, 2013).

Little is known about the role of K_V_ channels in erectile function (Archer, 2002). Although K_V_ channels are present and functional in CC (Christ et al., 1993; Malysz et al., 2001; Werner et al., 2005), their role in erection is not well elucidated. In human and mouse CC, both the delayed rectifier and the fast transient A K^+^ currents have been observed (Christ et al., 1993; Werner et al., 2005) while only the delayed rectifier current have been detected in rabbit CC. Delayed rectifier K^+^ channels appear to be important for the regulation and control of the membrane potential in cavernous smooth muscle cells (Malysz et al., 2001). In CC, KV2, and KV7 was shown to contribute to the voltage-dependent K^+^ currents (Malysz et al., 2002; Jepps et al., 2016). Targeting K^+^ channels can be considered a viable option for developing a treatment for ED that bypasses the NO/cGMP system and thus overcomes one of the major drawbacks of PDE5 inhibitors (Gopalakrishnan and Shieh, 2004; Hannigan et al., 2016).

Unjustifiably, most of the studies conducted to investigate K^+^ channels in the CC considered the (BK_Ca_) and K_ATP_ channels the most physiologically relevant (Lee, 2000; Andersson, 2011) and neglected the role of K_V_ channels in erection. Therefore, little is known about the role of K_V_ channels in erectile function**;** the current study thus aimed first at updating the knowledge about the effect of K_V_ channels in CC and secondly, to understand their role in NO action and/or synthesis.

## Materials and methods

### Chemicals

The chemicals used were 4-aminopyridine (4-AP), Atropine, Acetylcholine (ACh), Barium chloride (BaCl_2_), Glibenclamide, Guanethidine, N^w^ nitro-L-arginine (L-NNA), Phenylephrine (PE), Sodium nitroprusside (SNP), and Tetraethylammonium (TEA); all purchased from Sigma. All chemicals were dissolved in saline except Glibenclamide that was dissolved in polyethylene glycol (PEG) 400.

### Animals

Adult sexually mature male New Zealand White rabbits weighing 2.5–3 kg and male albino rats weighing 250–300 g were used. The animals were obtained from the Faculty of Pharmacy Alexandria University Animal House. All protocols adhere to international Animal Care and Use guidelines and approved by Faculty of Pharmacy—Alexandria University ethical committee (Protocol number ACUC 16/4).

### Organ bath experiments

#### The isolated rabbit corpus cavernosum

After rabbit exsanguination, the penis were excised rapidly and placed in Krebs solution at 4°C. A ventral incision was made on the right and left corpora, the tunica was dissected and the rabbit CC tissue was exposed. The corpora were dissected and subsequently studied in organ chambers. Strips of rabbit CC were tied at each end with cotton threads and mounted in a 25 ml organ chamber. Tissue baths containing Krebs solution were kept at 37°C and constantly bubbled with 95% O_2_ and 5% CO_2_. Before the start of the experiment, atropine (1 × 10^−6^M) and guanethidine (5 × 10^−6^M) were added to the organ bath to block muscarinic receptors and prevent the release of norepinephrine, respectively, during subsequent EFS. The upper part of each strip was attached to a force displacement transducer (Grass FT-03), which was connected through an MLAC11 Grass adapter cable to a computerized data acquisition system with Lab Chart-7 pro software (Power Lab 4/35, model ML 866/P; AD Instrument Pty Ltd, Castle Hill, Australia). The initial resting tension was 1 g. The preparation was left to equilibrate for 60 min. Each strip was submaximally contracted with PE (3 × 10^−6^ M). After the PE contractile response has stabilized, relaxation responses to different treatments were recorded in a cumulative fashion, or subjected to EFS-induced relaxation at supramaximal voltage (0.8 mspulse duration) using sequential frequencies of 2, 4, 8, and 16 Hz delivered as 10 s. trains. The relaxation responses were assessed as a percentage of the PE-induced contractile response. A time-matched control was performed regularly by adding saline (or PEG400 in case of glibenclamide) instead of the test drug to ensure stability of response along the duration of the experiment. Neither saline nor PEG400 in the volume added to the organ bath (0.8%) showed any variation in tone or response curves.

#### The isolated rat anococcygeus muscle

The muscle was prepared using male albino rats weighing 250–300 g according to the method described in 1972 by Gillespie (1972). The abdomen was opened and the bladder and the urethra were removed, the pelvis was raised with forceps and split along the midline using a pair of scissors. The bone was forced apart to reveal the terminal colon. The colon was lifted and the surrounding connective tissue was removed carefully toward the anus until the two anococcygeus muscles could be identified. The muscles arise from the vertebrae and can be followed to an angle to their point of meeting over the terminal colon. Each muscle was cleared from the surrounding connective tissue, tied at each end with cotton threads and mounted in a 25 ml organ chamber containing Krebs solution kept at 37°C and constantly bubbled with 95% O_2_ and 5% CO_2_. The initial resting tension was 1 g. The preparation was left to equilibrate for 30 min. EFS-induced relaxation was computed at supramaximal voltage (0.8 ms pulse duration) using sequential frequencies of 2, 4, 8, and 16 Hz delivered as 10 s. trains. Same data acquisition system was used as above-mentioned.

### Statistical analysis

The results obtained are expressed as mean ± SEM. Throughout the manuscript, numbers between parentheses indicate number of animals. The student-*t*-test was used for the analysis of paired/unpaired data whenever applies. The analysis of variance (ANOVA) followed by Bonferroni's post-test was used for multiple comparisons. The criterion for statistical significance was set at *p* < 0.05.

## Results

### Effect of potassium channel blockade on corpus cavernosum relaxation *In vitro*

**Figure 1:**
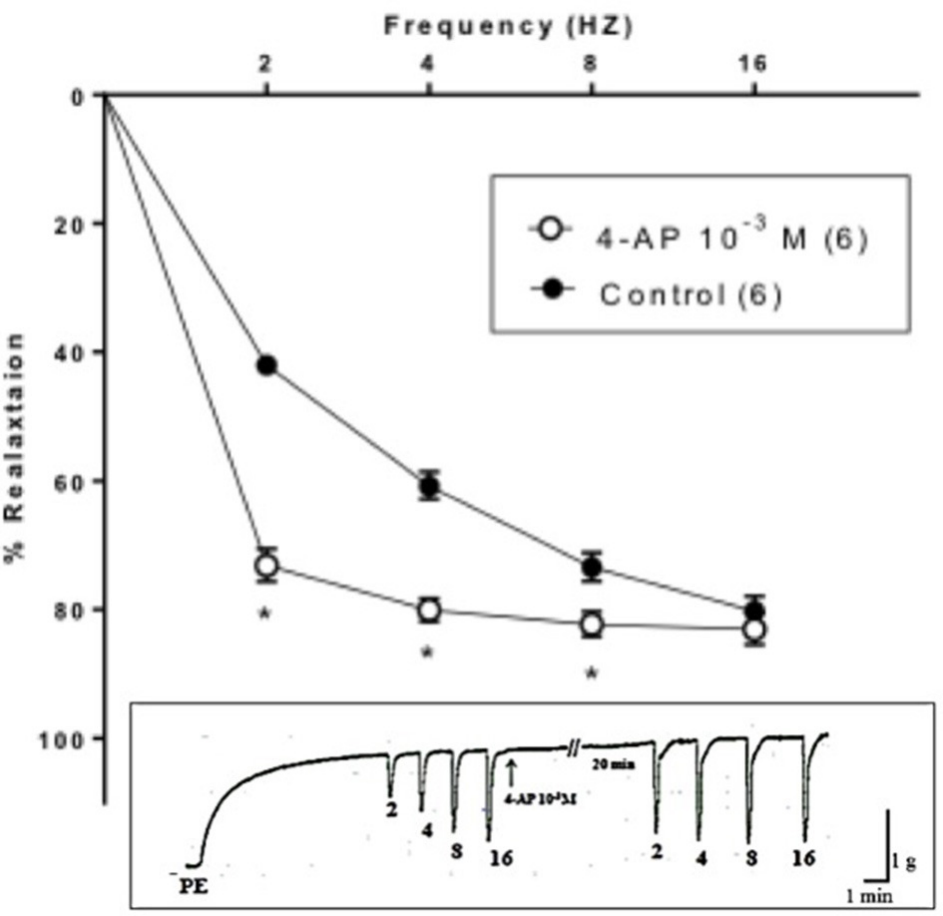
**Electric field stimulation-induced relaxation of phenylephrine precontracted rabbit corpus cavernosum in absence and in presence of 4-aminopyridine (4-AP, 10^**−3**^ M)**. Results are expressed as mean ± SEM of 6 animals. ^*^Denotes significant difference compared to control at the level of *P* < 0.05.

Strips of rabbit CC showed contractile responses induced by PE (3 × 10^−6^ M). Application of EFS elicited a transient frequency dependent relaxation response that increased in magnitude over a range of 2–16 Hz. 4-AP (10^−3^ M) significantly enhanced the EFS-induced relaxation responses of rabbit CC. A tracing is represented in Figure 1 showing the effect of 4-AP (10^−3^ M) on EFS-induced relaxation of rabbit CC. At 2 Hz, EFS-induced relaxation amounted to 73.17 ± 2.55% in presence 4-AP (10^−3^ M) compared to 41.98 ± 1.45% as control Figure 1.

**Figure 2:**
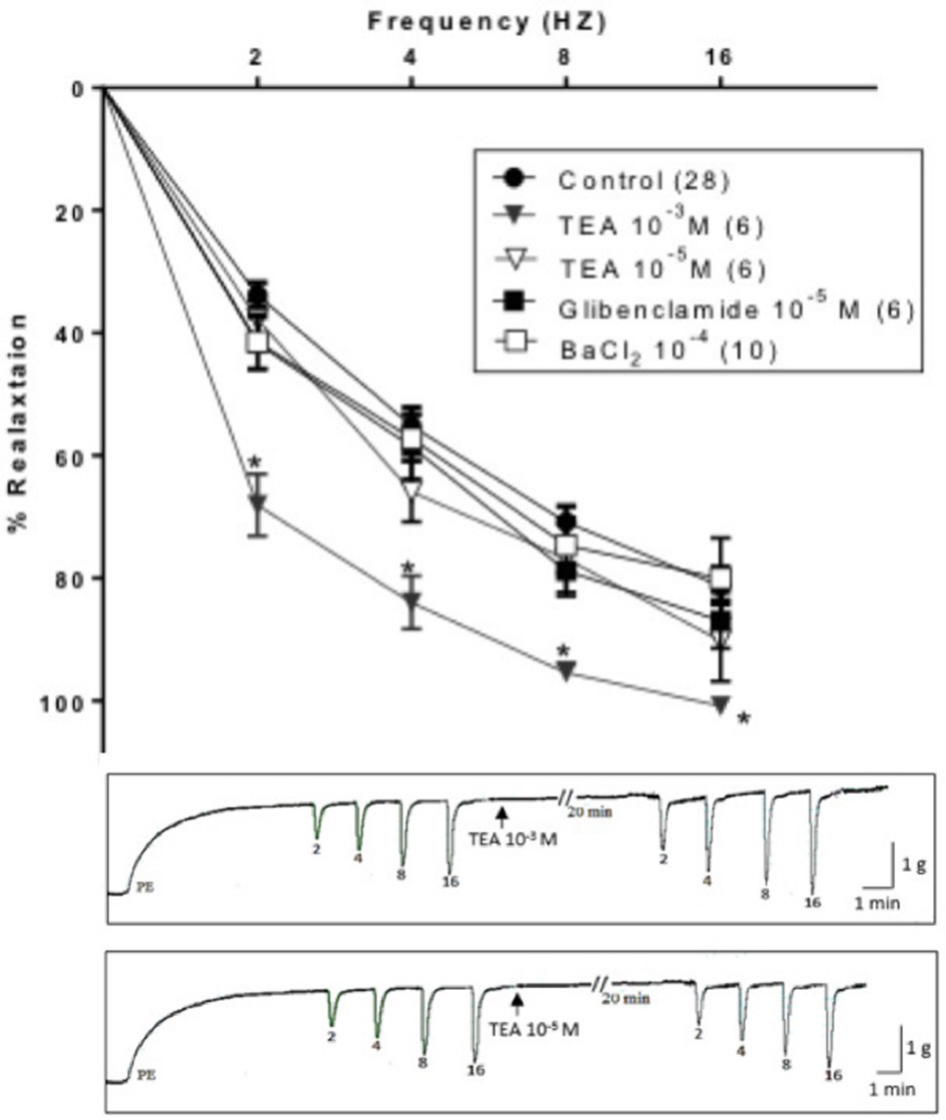
**Electric field stimulation-induced relaxation of phenylephrine precontracted rabbit corpus cavernosum in absence and in presence of tetraethylammonium (TEA, 10^−3^ M), TEA (10^−5^ M), glibenclamide (10^−5^ M) or barium chloride (BaCl_2_, 10^−4^ M)**. Results are expressed as mean ± SEM. Values between parentheses indicate the number of animals. ^*^Denotes significant difference than control at the level of *P* < 0.05. The representative tracings attached show the effect of TEA (10^−3^ M, upper panel) and TEA (10^−5^ M, lower panel).

Similar to 4-AP, TEA (10^−3^ M, non-selective concentration) potentiated EFS-induced relaxation. On the other hand, TEA (10^−5^ M, selective selective (BK_Ca_) blocker), glibenclamide (10^−5^ M, selective K_ATP_ blocker) and BaCl_2_ (10^−4^ M, selective Kir blocker) did not produce a significant change in the EFS-induced relaxation responses of rabbit CC Figure 2.

**Figure 3:**
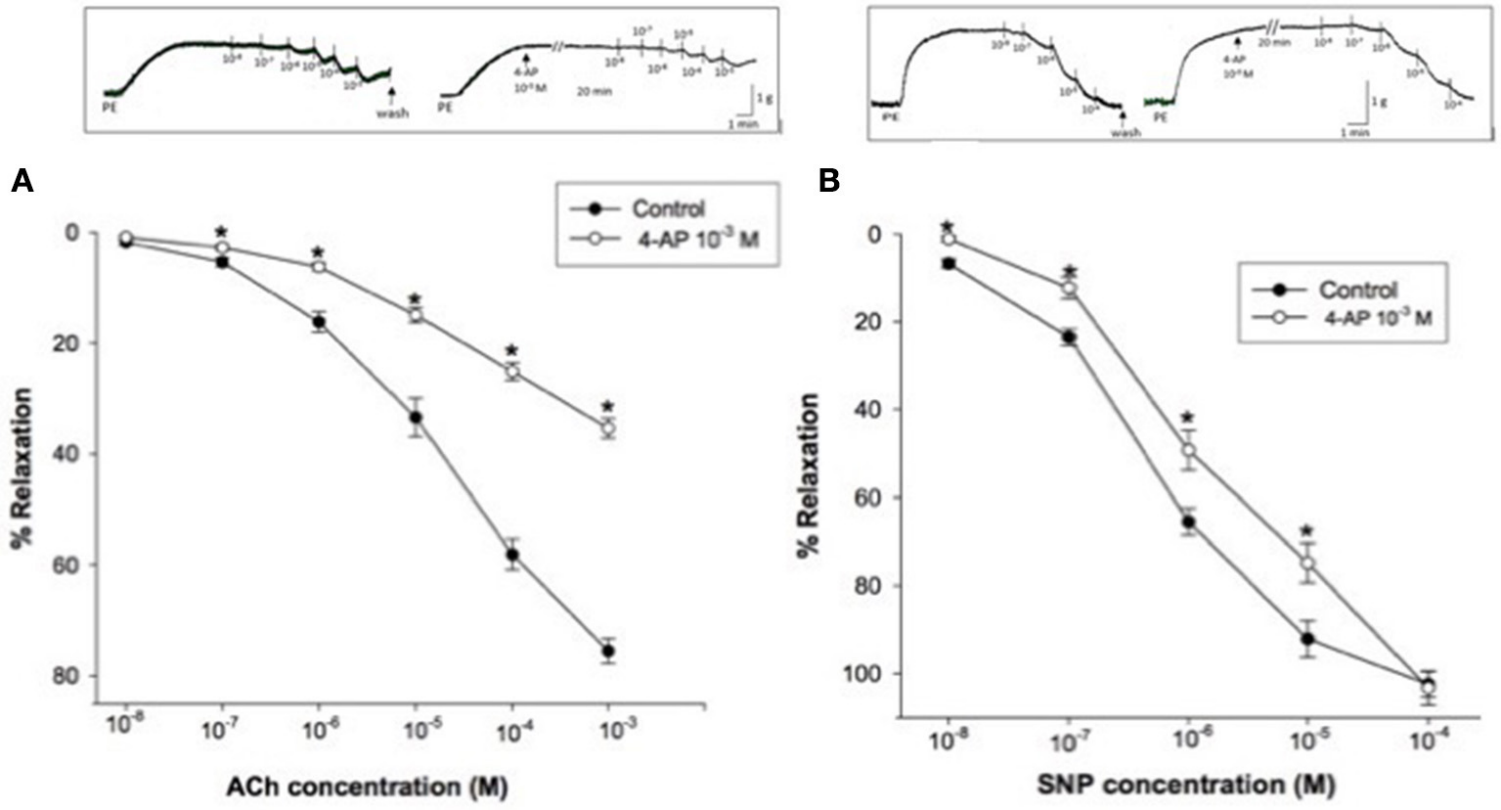
**(A)** Acetylcholine (ACh)-induced relaxation of phenylephrine precontracted rabbit corpus cavernosum in absence and in presence of 4-aminopyridine (4-AP, 10^−3^ M). Results are expressed as mean ± SEM of 8 animals. ^*^Denotes significant difference compared to control at the level of *P* < 0.05. **(B):** Sodium nitroprusside (SNP)-induced relaxation of phenylephrine precontracted rabbit corpus cavernosum in absence and in presence of 4-aminopyridine (4-AP, 10^−3^ M). Results are expressed as mean ± SEM of 9 animals.^*^Denotes significant difference compared to control at the level of *P* < 0.05.

Using PE (3 × 10^−6^ M) precontracted rabbit CC strips, a dose response curve for ACh (10^−8^–10^−3^ M) or SNP (10^−8^–10^−4^ M) was constructed. 4-AP (10^−3^M) significantly attenuated ACh-induced relaxation of rabbit CC where concentration-response curve was clearly shifted upward Figure 3A. Similarly, 4-AP (10^−3^M) significantly attenuated SNP- induced relaxation, for example, to 49.28 ± 4.52% compared to 65.53 ± 3.01% as control at 10^−6^ M SNP (Figure 3B).

### Interaction between potassium channels blockade and nitric oxide synthesis

#### Effect of tetraethylammonium (10^−3^M) and 4-aminopyridine (10^−3^M) on electric field stimulation-induced relaxation in presence of L-NNA in rabbit corpus cavernosum

Two concentration of the non-selective NOS blocker L-NNA have been used in this set of experiments. Both concentration (10^−5^ M or 2 × 10^−4^ M) significantly inhibited EFS-induced relaxation responses at all frequencies tested. At 2 Hz L-NNA (10^−5^ M) and (2 × 10^−4^ M) resulted in inhibition of relaxation corresponding to 61.68 ± 4.88% and 98.21 ± 0.52 respectively.

**Figure 4:**
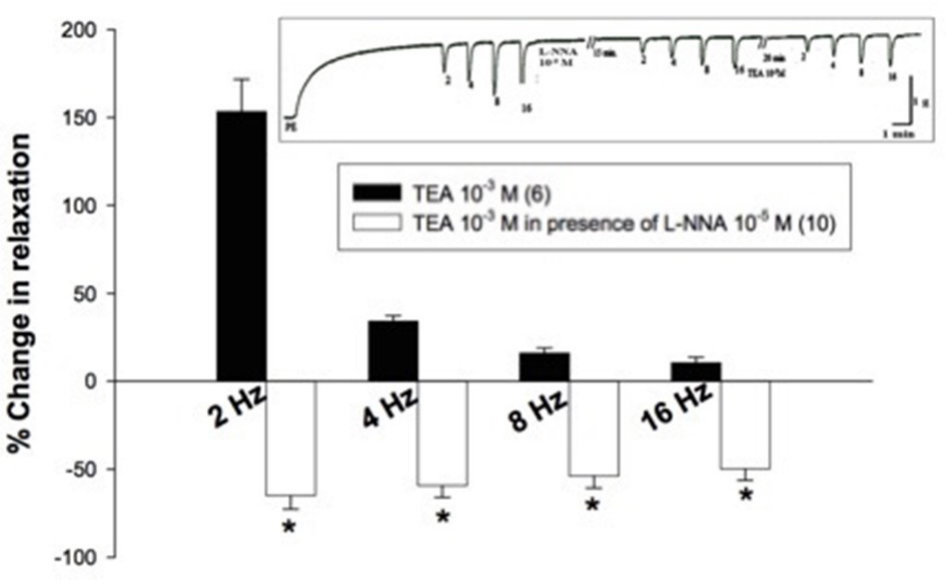
**Effect of tetraethylammonium (TEA, 10^−3^ M) on electric field stimulation- induced relaxation of phenylephrine precontracted rabbit corpus cavernosum in absence and presence of L-NNA (10^−5^ M)**. Results are expressed as mean ± SEM. Values between parentheses indicate the number of animals. ^*^Denotes significant difference compared to TEA (10^−3^ M) at the level of *p* < 0.05. L-NNA was added to the organ bath 15 min. prior to the addition of TEA.

**Figure 5:**
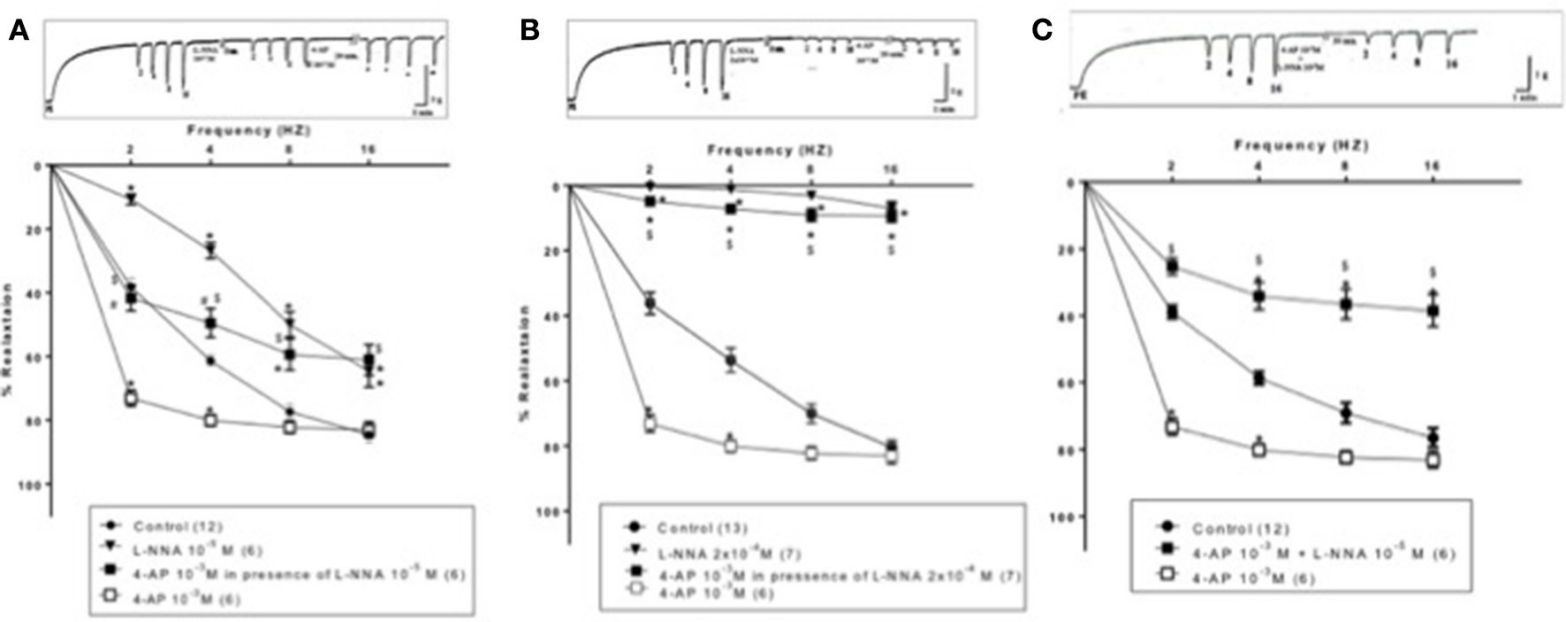
**Effect of 4-aminopyrdine (4-AP, 10^−3^ M) on electric field stimulation-induced relaxation of phenylephrine precontracted rabbit corpus cavernosum in absence and presence of L-NNA (10^−5^ or 2 × 10^−4^ M)**. **(A)**: L-NNA (10^−5^) was added to the organ bath 15 min prior to 4-AP addition. **(B)** L-NNA (2 × 10^−4^ M) was added to the organ bath 15 min prior to 4-AP addition. **(C)**: 4-AP and L-NNA (10^−5^ M) were added simultaneously to the organ bath. Results are expressed as mean ± SEM. Values between parentheses indicate the number of animals. ^*^Denotes significant difference compared to control at the level of *p* < 0.05. ^#^Denotes significant difference compared to L-NNA at the level of *p* < 0.05. ^$^denotes significant difference compared to 4-AP (10^−3^ M) at the level of *p* < 0.05.

TEA (10^−3^ M) added to the organ bath after 15-min incubation with L-NNA (10^−5^ M) caused inhibition of EFS-induced relaxations rather than potentiation (Figure 4). Figure 5, on the other hand, shows the same experiment using the selective K_V_ blocker, 4-AP instead of TEA. 4-AP (10^−3^ M) in presence of L-NNA (10^−5^ M, incubated for 15 min) potentiated relaxations at 2 and 4 Hz only when compared to L-NNA effect alone. Its effect at 8 and 16 Hz was significantly lower than control indicating an inhibition of its potentiatory action (Figure 5A).

When the same protocol was repeated using a higher concentration of L-NNA (2 × 10^−4^ M, incubated for 15 min), the potentiatory effect of 4-AP on EFS- induced relaxation responses was masked at all frequencies tested. Relaxation induced by EFS after 4-AP showed no significance vs. L-NNA alone but was highly significantly lower than 4-AP effect and control as well (Figure 5B). For example, at 2 Hz, 4-AP (10^−3^ M) in presence of L-NNA (2 × 10^−4^ M) inhibited control EFS-induced relaxation by 78.50 ± 6.43% (*n* = 7), compared to L-NNA alone which inhibited relaxation by 98.21 ± 0.52% (*n* = 7). 4-AP alone potentiated the relaxation at the same frequency by 74.86 ± 6.46% (*n* = 6). In another experimental design, 4-AP (10^−3^M) and L-NNA (10^−5^ M) were added simultaneously to the organ bath (Figure 5C), the combination effect was significantly lower than control and 4-AP alone at 2,4,8, and 16 Hz.

#### Effect of 4-aminopyridine in absence and in presence of L-NNA on electric field stimulation-induced relaxation in presence of L-NNA in rat annococcygeus muscle

**Figure 6:**
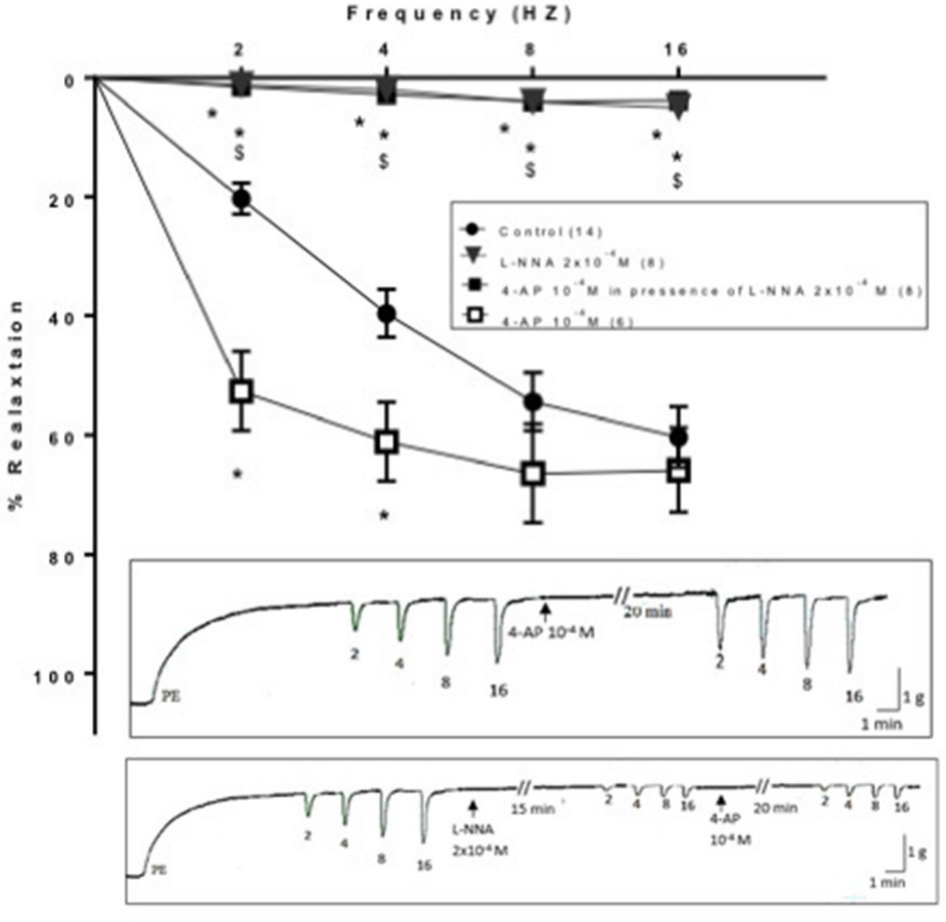
**Effect of 4-aminopyrdine (4-AP, 10^−4^ M) on electric field stimulation-induced relaxation of phenylephrine precontracted rat anococcygeus muscle in absence and presence of L-NNA (2 × 10^−4^ M)**. L-NNA (2 × 10^−4^ M) was added to the organ bath 15 min. prior to 4-AP addition. Results are expressed as mean ± SEM. Values between parentheses indicate the number of animals. ^*^Denotes significant difference compared to control at the level of *p* < 0.05. ^#^Denotes significant difference compared to L-NNA at the level of *p* < 0.05. ^$^Denotes significant difference compared to 4-AP (10^−4^ M) at the level of *p* < 0.05.

The isolated rat annococcygeus muscle showed contractile responses induced by PE (3 × 10^−6^ M). Application of EFS elicited a transient frequency dependent relaxation response that increased in magnitude over a range of 2–16 Hz. Blockade of NOS by L- NNA (2 × 10^−4^ M) produced a reduction in the EFS-induced relaxation responses of rat anococcygeus muscle. At 2 Hz, the percentage relaxation induced by EFS in presence of L-NNA (2 × 10^−4^ M) was 1.16 ± 0.34 compared to 22.99 ± 3.4 as control. 4-AP (10^−4^ M) significantly enhanced the EFS-induced relaxation responses of rat annococcygeus muscle at 2 and 4 Hz causing a downward shift of the frequency response curve (Figure 6). In combination, and when 4-AP (10^−4^ M) was tested in presence of L-NNA (2 × 10^−4^ M, incubated for 15 min), its effect was totally abolished; it failed to potentiate relaxation induced by EFS at all frequencies tested. The frequency response curves in presence of L-NNA and the combination were almost superimposed and significantly lower than control (Figure 6).

## Discussion

The selective role of different types of K^+^ channels as modulators of penile erection is under investigated. Additionally, the relative contribution of the different types of K^+^ channels to relaxations induced by NO-donors and ACh in rabbit CC has not been fully elucidated. Therefore, the first aim of the present study was to update the information about K_V_ channels in CC and its contribution to the relaxations induced by ACh and NO in CC and secondly, to investigate the possible interactions between K_V_ channels and NO in CC. In the present study, treatment of rabbit CC with the K_V_ blocker, 4-AP, reduced the SNP and ACh-induced relaxations, an effect which indicates a role for K_V_ channels in mediating NO action. The blockade of K_V_ channels also attenuated SNP and ACh-induced relaxations in rat aorta (Satake et al., 1997; Fiorim et al., 2012; Dias et al., 2014; Oliveira et al., 2014). In line with these observations, K_V_ channels have been implicated in the ACh-induced increase in ICP in cats as described by Moon et al. (1999). Moreover, 4-AP was reported as a possible allosteric modulator of muscarinic receptors in rat brain (Lai et al., 1985). In contrast, in a study by Andre et al. (2003) 4-AP did not reduce the relaxations induced by ACh in rabbit CC. This discrepancy may be attributed to the difference in the conditions of the experiment where in the latter study, indomethacin was added to the Krebs solution. Based on the limited number of studies in literature and the opposing observations, it seems that the role of K_V_ channels in ACh-evoked endothelium-dependent relaxations is under investigated in CC. Concerning EFS-induced relaxation, in the present study 4- AP surprisingly potentiated EFS-induced relaxation responses. The same effect was produced by the non-selective K^+^ channels blocker TEA (10^−3^M). This potentiatory effect is unexpected since—as described by Malysz and co-workers- K_V_ currents increased excitation of rabbit CC myocytes, and hence should have enhanced contraction or reduce relaxation (Malysz et al., 2002). Although neither the design of this study nor the dose of 4-AP used targeted the investigation of a potential effect on contraction, the unexpected potentiation of relaxation induced by 4-AP was studied in depth by *in vitro* experiments using rabbit CC and rat anococcygeus muscle- which is part of the erectile machinery in male rodents (Gillespie, 1972). To our knowledge the current study is the first to demonstrate that the blockade of the K_V_ channels by 4-AP possess a potentiatory effect on the EFS-induced relaxation responses in rabbit CC. These results are in agreement with studies performed in other tissues including: canine iloecolonic junction (De Man et al., 1993), lamina propria of the female rabbit urethra (Zygmunt et al., 1996), sheep urethra (Costa et al., 2001), and pig urinary bladder neck (Hernandez et al., 2008). All these studies suggested a prejunctional modulatory role of K_V_ channels in nitrergic neurotransmission. Since treatment of rabbit CC with 4-AP reduced the SNP-induced relaxation, the latter observation provides further evidence that the potentiation of EFS- induced relaxation by 4-AP is mediated via a pre-junctional and not a post-junctional effect. It is to be mentioned that only the non-selective and 4-AP, the selective K_V_ channel blockers potentiated EFS-induced relaxation in contrast to other channels blockers tested.

A hypothesis was put forward by Hernandez et al. (2008) to explain this potentiation in the pig urinary bladder neck. Arrival of action potentials at the nitrergic nerve terminal evokes membrane depolarization and activation of voltage-gated Ca^2+^channels with the subsequent Ca^2+^ influx. Increased cytosolic Ca^2+^ stimulates nNOS through interaction with calmodulin, and would favor NO synthesis from L- arginine and release from nerves. In addition to the opening of voltage-gated Ca^2+^channels, an activation of neuronal (pre-junctional) K_V_ channels downregulate the NO synthesis, probably through inhibition of voltage-gated Ca^2+^channels, thus increasing the hyperpolarizing post-potential phase (Hernandez et al., 2008). Therefore, the blockade of K_V_ channels probably prolong the depolarization phase and hence increase the synthesis of NO and eventually potentiate the EFS-induced relaxation. This hypothesis is supported by the finding that neurons express different types of K^+^ channels (Bowery and Smart, 2006). Furthermore, 4-AP has been found to increase neurotransmitter release in the CNS (Tibbs et al., 1989; Schnee and Brown, 1998; Luca and Singer, 2013; Li et al., 2016); an effect that also favors the proposed hypothesis. It is to be mentioned that the compound (4-AP) has been used in many studies of vascular smooth muscle as a K_V_ channel blocker in order to separate the K_V_ current from (BK_Ca_) current, which is also activated by membrane depolarization (Ko et al., 2008). In this context, the exclusion of the effect of (BK_Ca_) channels by adding iberiotoxin (the selective (BK_Ca_) blocker) to the organ bath, will be of value in future experiments to confirm the proposed role for K_V_ channels, since both K_V_ and (BK_Ca_) channels are activated by depolarization. It remains to point out that a sustained-release form of 4-AP which is also known by its international non-proprietary name, fampridine has been developed and is currently licensed for the treatment of walking impairment in multiple sclerosis (Krishnan and Kiernan, 2013).

To test if the previous hypothesis applies in rabbit CC, the effect of 4-AP and TEA (10^−3^M) on EFS-induced relaxation was tested in the presence of the non-selective NOS inhibitor L-NNA. In this context, the effect of TEA (10^−3^ M) on EFS-induced relaxation was tested in presence of L-NNA (10^−5^ M, a dose tested to produce 50% decrease in EFS-induced relaxation). Interestingly, in presence of L-NNA the potentiatory effect of TEA on EFS-induced relaxation was abolished. This was followed by examining the effect of 4-AP in presence of L-NNA. To accomplish this objective and obtain evidence about the proposed theory, this experiment was performed using 3 different protocols. In the first protocol, 4-AP and L-NNA (10^−5^ M) were added simultaneously to the organ bath and their effect on EFS-induced relaxation was tested according to the method described by Hernandez et al. (2008). In the second and third protocols, either L- NNA (10^−5^ M) or (2 × 10^−4^ M, tested to produce 98% reduction in EFS-induced relaxation) were added to the organ bath followed by testing the effect of 4-AP. In all 3 protocols, 4- AP did not potentiate EFS-induced relaxation responses after NOS blockade, and same observation was observed and confirmed in the anococcygeus muscle which is a part of the erectile machinery in the rat. These results are similar to that obtained by Hernandez et al. (2008) in the pig urinary bladder neck, which provides evidence that the blockade of neuronal K_V_ channels did increase NO synthesis and therefore resulted in the potentiation of EFS-induced relaxation. Consequently, this potentiation was abolished upon the inhibition of NO synthesis by L-NNA. As mentioned earlier in this discussion, 4-AP inhibited ACh-induced relaxation (even more than SNP relaxation); therefore, we cannot rule out the presence of K_V_ channels equally in endothelium, the blockade of which may have caused a reduction in synthesis of ACh-dependent relaxing factors. Unlike smooth muscle cells, endothelial cells lack voltage gated Ca^+2^ channels and are electrically non-excitable, and hyperpolarization will tend to increase intracellular Ca^+2^ concentration by enhancing the electrochemical gradient that drives transmembrane Ca^+2^ influx in the endothelium. This increase of intracellular Ca^+2^ concentration is critical for the release of endothelial relaxing factors such as NO, EDHF and prostacyclin (Kamouchi et al., 1999; Triggle et al., 2012). Further experiments using specific nNOS inhibitor may be required to further elucidate these results, as well as immunohistochemistry studies to compare localization of K_V_ channels between nerves and endothelial smooth muscle cells.

## Conclusion

In conclusion, it seems that K_V_ channels may have a pre-junctional role in modulating NO synthesis in corpus cavernosum, where their blockade may increase NO synthesis.

## Author contributions

AS contributed to the design of the experiments, their execution, and analysis of data, interpretation of data and discussion of the results as well as to the revision of figures and editing of the manuscript. HA conducted efficiently the organ bath experiments, contributed to the experimental design and analysis of data. She was responsible for the literature review and the original editing of the manuscript. FS contributed to the design of the experiments, interpretation of data and discussion of the results as well as to the revision of figures and the whole manuscript. MM contributed to the development of the original idea of the manuscript, review and analysis of data, interpretation and discussion of the results as well as revision of the manuscript.

### Conflict of interest statement

The authors declare that the research was conducted in the absence of any commercial or financial relationships that could be construed as a potential conflict of interest.
